# Flexor digitorum longus tendon transfer to the navicular: tendon-to-tendon repair is stronger compared with interference screw fixation

**DOI:** 10.1007/s00167-018-4936-0

**Published:** 2018-04-07

**Authors:** Daniel Marsland, Joanna M. Stephen, Toby Calder, Andrew A. Amis, James D. F. Calder

**Affiliations:** 1 Fortius Clinic, 17 Fitzhardinge St, London, W1H 6EQ UK; 2 The Biomechanics Group, Department of Mechanical Engineering, Imperial College London, London, UK; 3 Winchester College, Winchester, UK; 4 Musculoskeletal Surgery Group, Department of Surgery and Cancer, Imperial College London School of Medicine, London, W6 8RF UK

**Keywords:** Tendon transfer, Pes planus, Tibialis posterior tendon dysfunction, Navicular, Interference screw, Flexor digitorum longus

## Abstract

**Purpose:**

To assess whether early rehabilitation could be safe after flexor digitorum longus (FDL) tendon transfer, the current biomechanical study aimed to measure tendon displacement under cyclic loading and load to failure, comparing a traditional tendon-to-tendon (TT) repair with interference screw fixation (ISF).

**Methods:**

24 fresh-frozen cadaveric below knee specimens underwent FDL tendon transfer. In 12 specimens a TT repair was performed via a navicular bone tunnel. In a further 12 specimens ISF was performed. Using a materials testing machine, the FDL tendon was cycled 1000 times to 150 N and tendon displacement at the insertion site measured. A final load to failure test was then performed. Statistical analysis was performed using two-way ANOVA and an independent *t* test, with a significance level of *p* < 0.05.

**Result:**

No significant difference in tendon displacement occurred after cyclic loading, with mean tendon displacements of 1.9 ± 1.2 mm (mean ± SD) in the TT group and 1.8 ± 1.5 mm in the ISF group (n.s.). Two early failures occurred in the ISF group, none in the TT group. Mean load to failure was significantly greater following TT repair (459 ± 96 N), compared with ISF (327 ± 76 N), *p* = 0.002.

**Conclusion:**

Minimal tendon displacement of less than 2 mm occurred during cyclic testing in both groups. The two premature failures and significantly reduced load to failure observed in the ISF group, however, indicate that the traditional TT technique is more robust. Regarding clinical relevance, this study suggests that early active range of motion and protected weight bearing may be safe following FDL tendon transfer for stage 2 tibialis posterior tendon dysfunction.

## Introduction

Flexor digitorum longus (FDL) tendon transfer is an important component of the surgical treatment for stage 2 posterior tibialis tendon dysfunction [3, 14, 19, 24]. It is most commonly performed by suturing the FDL tendon back on to itself via an intraosseous tunnel through the navicular, known as a tendon-to-tendon (TT) repair [3, 17, 22, 24, 25, 28]. Interference screw fixation (ISF) has emerged as an alternative method with potential advantages including use of a shorter tendon graft and less surgical dissection compared with the standard TT technique [2, 4, 30]. Prior to the development of foot-specific hardware, large diameter bioabsorbable screws designed for anterior cruciate ligament reconstruction (ACL) were used in the foot requiring oversized pilot holes relative to the FDL tendon diameter [5, 17, 25].

At present postoperative protocols after FDL tendon transfer typically employ non weightbearing cast immobilisation for 4–6 weeks [4, 30], which may contribute to stiffness, muscle atrophy and venous thromboembolism (VTE). Earlier weight bearing and range of motion would potentially improve patient satisfaction, neuromuscular control and reduce the duration of chemical VTE prophylaxis [13, 20]. The main risk of early rehabilitation would be tendon slippage at the fixation site, leading to tendon lengthening and muscle weakness [10].

The optimal method of tendon fixation for FDL tendon transfer remains unclear, as are the effects of early tendon loading. Previous biomechanical work has shown that fixation with a 7 mm bioabsorbable interference screw had a significantly weaker pullout strength compared with the TT repair [25]. Smaller diameter interference screws have since become available, allowing smaller pilot holes which match the tendon diameter, with promising early clinical results [4]. The current study aimed to compare traditional TT repair with ISF in a cadaveric foot model. The primary null hypothesis was that there would be no difference in tendon displacement under cyclic loading. The secondary null hypothesis was that there would be no difference in loads to failure.

## Materials and methods

Twenty-four cadaveric fresh-frozen below knee specimens aged 38.5 ± 9.5 (27–63) years (mean ± SD, range), with no history of disease or previous surgery were obtained from a tissue bank. Specimens consisting of whole feet and approximately 400 mm of tibia, were stored in a freezer at − 2 0 °C before use and thawed on the day of experimentation. Testing on each specimen took place in a single day.

Using a medial incision in the foot, the posterior tibialis tendon was released from its insertion into the navicular and excised. The FDL tendon was then identified and sectioned at the master knot of Henry, obtaining as much tendon length as possible. The tendon end was then prepared using a 2/0 Ethibond whipstitch (Ethicon, Johnson and Johnson, NJ, USA).

**Fig. 1:**
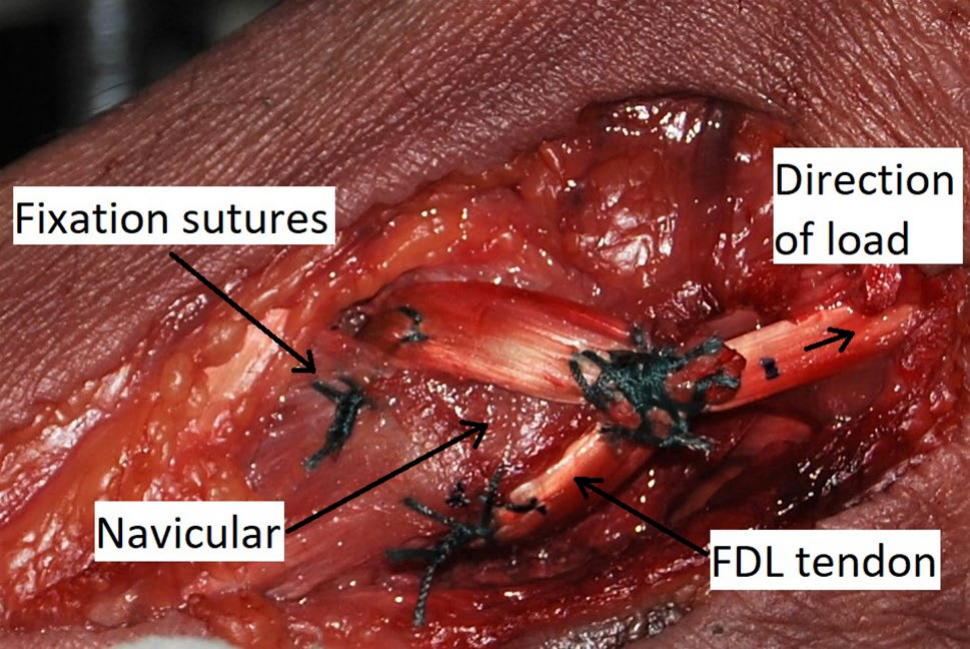
Medial view of a right foot specimen. Tendon-to-tendon suturing of FDL via a navicular bone tunnel

For the TT group (*n* = 12), a 4.5 mm bicortical bone tunnel was made in the navicular using a cannulated drill over a guidewire, from plantar to dorsal. The FDL tendon was then passed from plantar to dorsal. With tension applied as would be done in the clinical setting, the tendon end was sutured back on to itself proximally using two Ethibond sutures (Fig. 1). Further sutures were also placed at the plantar and dorsal entrances to the bone tunnel securing the tendon to the periosteum. One specimen was chosen for a pilot test on which only a load to failure test was performed; this left 11 specimens for cyclic testing.

**Fig. 2:**
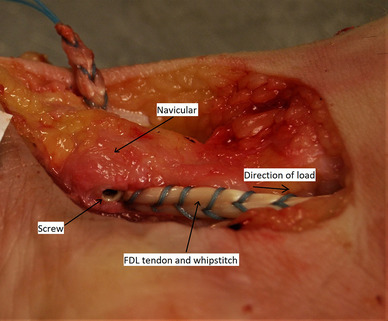
Plantar medial view of a right foot specimen. The FDL tendon has been prepared with a whipstitch and then fixed with an interference screw, inserted in the plantar aperture of the navicular bone tunnel

For ISF (*n* = 12), the tendon diameter was measured using sizing sleeves in 0.5 mm increments. All tendons measured 4 or 4.5 mm. A bicortical bone tunnel was made from plantar to dorsal, ensuring the tunnel diameter matched the FDL tendon diameter. The FDL tendon was pulled through the bone tunnel from plantar to dorsal and tensioned. A 4.75 mm × 15 mm interference screw (Fig. 2) was then inserted into the plantar aperture as per the manufacturer’s guidelines (PEEK Bio-Tenodesis screw, Arthrex, Naples, FL, USA). For all specimens, the wound was left open, to allow the tendon-bone motion to be observed during testing.

Following completion of the tendon transfer, each foot was placed in a custom made aluminium tray taking care to ensure the hindfoot was in a neutral position. Each foot was then fixed in place to the tray using two Steinmann pins through the calcaneum and one Steinmann pin across the forefoot via the metatarsals. The tray was then filled with acrylic cement, encasing the foot to prevent sliding within the tray.

Using a marker pen, markings were made on the FDL tendon and at the insertion site on the navicular, allowing subsequent measurement of tendon displacement relative to the insertion point.

### Specimen testing

**Fig. 3:**
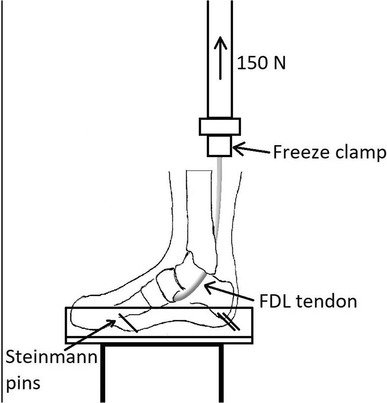
Specimen set up. The foot was fixed into a custom made aluminium tray using Steinmann pins and acrylic cement. The FDL tendon was fixed to the hydraulic arm of the Instron machine using a freeze clamp and then loaded

Specimens were loaded in an Instron 8874 servo-hydraulic materials testing machine (Instron, High Wycombe, UK). The FDL tendon was identified proximally at the musculotendinous junction and sectioned. The proximal tendon end was then held using an established technique in a freeze clamp attached to the actuator of the testing machine [23]. The specimen was positioned on the machine under the actuator so that the load would be applied in line with the physiological line of pull of the FDL tendon (Fig. 3).

The FDL tendon was preloaded to 100 N (ramp rate 10 N/s for 10 s). Baseline digital photographs were taken of the tendon insertion point. Cyclic loading from 50 to 150 N was then commenced at a rate of 1 Hz, for 1000 cycles. Digital photographs were taken every 100 cycles until completion of testing. For each photograph a ruler was placed next to the wound so that accurate scale measurements of tendon-bone displacements could be made using a digital software program [12]. Following cyclic loading, a load to failure test was performed, at a rate of 1000 mm per minute.

Ethics approval was obtained from the Tissue Management Committee of Imperial College Healthcare Tissue Bank (Application No. R17009).

### Statistical analysis

Using load to failure as the endpoint, to show a difference of 131 ± 81 N between groups, power analysis (*p* = 0.05, power 0.80) showed that seven specimens would be required in each group [25]. The current study did not use matched pairs, so the sample size was increased to 12 specimens in each group. Two-way ANOVA was performed to compare displacement of the tendon transfer during cyclic loading for each technique. To determine measurement error two authors (DM and TC) independently took a sample of 50 measurements, with one author (TC) repeating each measurement. Inter- and intra-rater reliability for measurement of tendon displacement were subsequently assessed using two-way mixed, absolute agreement, average measures intraclass correlation coefficients. An independent samples *t* test was used to compare mean loads to failure. Data were analysed in SPSS (IBM SPSS Statistics for Windows, version 24.0; IBM, Armonk, NY), with significance set at *p* < 0.05.

## Results

### Cyclic testing

**Fig. 4:**
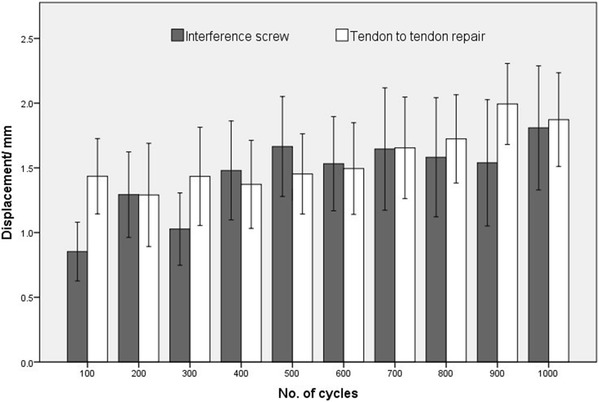
Graph of tendon displacement during cyclic loading (mean ± SE; *n* = 12 for interference screw group and *n* = 11 for tendon-to-tendon repair group)

All 11 specimens in the TT group survived cyclic testing, during which the mean tendon displacement was 1.9 ± 1.2 (0.9–4.0) mm. In the ISF group, 10 out of 12 specimens survived cyclic testing, with a mean tendon displacement of 1.8 ± 1.5 (0.0–4.7) mm. There was no significant difference among the constructs that survived the testing, when comparing fixation methods (n.s.). Tendon displacement compared with baseline was statistically significant in both groups (*p* < 0.001; Fig. 4).

In the two specimens that failed during cyclic loading, failure occurred after 100 and 500 cycles, respectively. In the first specimen (31-year-old male), the screw appeared to have partially backed out by approximately three threads. The FDL tendon in this specimen had a 4.5 mm diameter with a 4.5 mm bone tunnel, and there were no technical difficulties during screw insertion. In the second specimen (44-year-old male), the tendon diameter was 4 mm with a matching bone tunnel. It was noted in this specimen that screw advancement was difficult, possibly due to hard bone and a slightly oversized screw. Post-testing assessment revealed tunnel deformity (eccentric expansion), but no obvious screw damage.

Correlation coefficients for inter- and intra-rater reliability were 0.998 and 0.999, respectively, indicating excellent agreement in measurement of tendon displacement. The mean measurement error was 0.4 ± 0.4 (0.0–2.5) mm.

### Load to failure

**Fig. 5:**
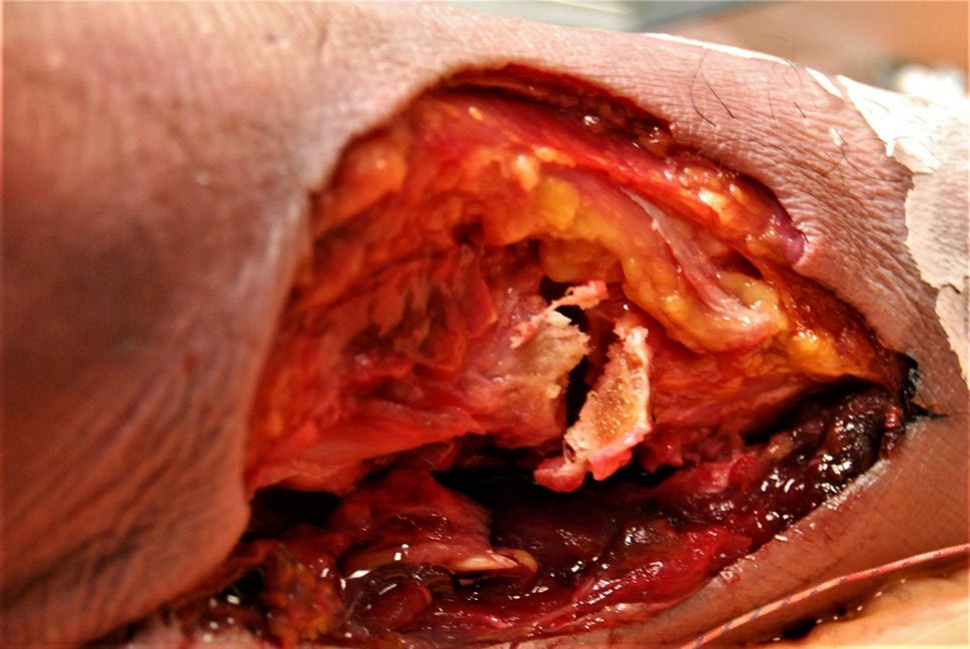
Medial view of a left foot specimen. Navicular tunnel fracture after a load to failure test in a specimen with a tendon-to-tendon repair

Load to failure in the TT group (*n* = 12) was 459 ± 96 (271 to 544) N, significantly greater in comparison to the ISF group at 327 ± 76 (148 to 415) N, *p* = 0.002. The mode of failure in the TT group was tunnel fracture in six specimens (Fig. 5), failure of the sutures in five and mid tendon rupture in one specimen. All specimens in the ISF group (*n* = 10) failed at the bone–screw interface; post-test examination revealed tunnel deformity in all specimens.

## Discussion

The most important finding of this study is that for FDL tendon transfer, a traditional TT repair is more robust compared with ISF. During cyclic loading which simulated early mobilisation there was no significant difference in mean tendon displacement comparing techniques. There were, however, two early failures in the ISF group. In addition, load to failure was significantly weaker in the ISF group compared with the TT repair, rejecting the null hypothesis that the techniques are comparable. The results suggest that TT repair is the more reliable surgical technique, and that it may have sufficient strength to withstand the cyclic loads associated with early protected weight bearing and mobilisation. Early rehabilitation is desirable compared with plaster immobilisation following FDL tendon transfer, with potential benefits including improved patient satisfaction, less stiffness, reduced risk of VTE and shorter duration of chemical VTE prophylaxis [13, 20].

The specimens were subjected to 150 N cyclic loading to represent physiological loads expected during early postoperative protected weight bearing, and non weightbearing active ankle plantarflexion [1, 6, 11, 17]. It has been suggested that the critical initial fixation strength required for an FDL tendon transfer is 50 N [11, 17]. More recent work, based on finite element modelling and measurement of muscle cross-sectional areas, estimated that a normal FDL muscle is likely to generate greater forces, up to 274 N [1, 6]. Considering that after FDL tendon transfer muscle strength would be likely to reduce, the mean loads to failure in this study of 459 N for TT repair and 327 N for ISF indicate that both may offer adequate initial pullout strength.

Although excessive tendon lengthening due to loss of fixation at the insertion site could lead to muscle atrophy and weakness [10], cyclical loading of FDL tendon transfers has not previously been reported. The current study found that mean tendon displacement was less than 2 mm in both TT and ISF groups, which we consider acceptable in the clinical setting. The potential disadvantage of ISF is early tendon pullout, which occurred in 2 out of 12 specimens. Early tendon pullout would be a major complication in a patient, and has been reported in other biomechanical work testing ISF in the foot. Drakos et al. simulated FHL transfer to the calcaneum, using a 5.5 mm diameter interference screw [7]. In that study, 3 out of 10 specimens failed within 100 cycles at loads of only 60 N [7].

TT fixation performed reliably during cyclic testing, with no failures and a high mean pullout strength. The most common mode of failure was tunnel fracture. This would seem an unlikely event in the clinical setting, especially if the foot were protected in a boot whilst full weightbearing, preventing sudden movements. Previous biomechanical studies that have assessed FDL tendon transfer fixation, report lower pullout strengths than we found, possibly due to differences in bone density (the mean age of our specimens was 38 years) [25, 28]. In osteoporotic specimens, Sabonghy et al. reported that TT fixation had a mean pullout strength of 279 N, significantly stronger than fixation using a 7 mm diameter bioabsorbable interference screw [25]. Sullivan et al. reported mean loads to failure of 142 N following both TT repair or FDL tendon transfer using suture anchors [28]. Although all specimens in that study were under 55 years of age, only two Ethibond sutures were used to perform the TT repair. Instead, we used additional sutures to secure the tendon to the periosteum at both the dorsal and plantar tunnel entrances, which is our standard surgical technique (Fig. 1).

Interference screw fixation (ISF) is well established in the knee [21, 29]. The foot in comparison presents some technical difficulties, including small diameter tendon grafts. In our study the FDL tendon consistently measured either 4 or 4.5 mm in diameter. It has been shown that initial pullout strength is maximised if the tunnel diameter matches the tendon diameter [27]. This was not possible in the foot previously because screws less than 5 mm diameter were not widely available and could not reliably tolerate high torque during insertion [8]. Therefore, oversized holes and, often, bioabsorbable screws normally used for ACL reconstruction were employed [5, 8, 17, 25]. In two previous biomechanical studies, pull out strengths for FDL transfer using 7 mm screws were 148 and 170 N [17, 25]. Since then, advances in screw design and use of materials such as PEEK have led to the routine use of small diameter screws. In the current study, although inferior to TT repair, the 4.75 mm ISF had a relatively high pullout force (327 N) compared with previous studies. This difference may be explained partly by the ability to match the pilot hole to the tendon diameter, differences in screw properties, the use of a whipstitch which has been found to increase fixation strength [16], and the use of younger specimens compared with previous studies [17, 25].

The in vitro nature of this study carries some limitations. Sample size was deemed adequate; post hoc analysis using load to failure showed the current study had a power of 0.94 to show a difference between techniques. Post hoc power analysis using tendon displacement was not possible, because the effect size was zero. This work simulated initial fixation strength but was unable to simulate the effects of tendon to bone healing expected to progress during the first 6 weeks [18]. We also only tested one screw diameter in the ISF group; the impact of a larger diameter PEEK screw is unknown. Although the cadaveric specimens were from relatively young adults, a range of bone density might be expected, but was not measured. We did not simulate a medial displacement calcaneal osteotomy, which is often performed in conjunction with an FDL tendon transfer. This osteotomy is inherently stable, with loads to failure for screw or step plate fixation of 947 and 1706 N, respectively [15]. Therefore, as reported by others [9, 26], we consider it safe to bear full weight within 4 weeks of surgery.

The clinical relevance of this study is that the mechanical data on repair strength and resistance to elongation under cyclic loading support the idea that a clinical study could find that shorter periods of cast immobilisation may be safe rather than the current standard period of 6 weeks following FDL transfer. As an illustration of what might be aimed for: the postoperative regime could include 2 weeks of cast immobilisation, and assuming satisfactory wound healing at that stage, conversion to a boot. Active nonweightbearing plantarflexion exercises could then be commenced, avoiding dorsiflexion exercises until 6 weeks. Depending upon whether additional procedures had been performed such as a calcaneal osteotomy, protected weightbearing in the boot could be started at 2–4 weeks.

## Conclusion

The current cadaveric study suggests that for TT repair following FDL tendon transfer to the navicular, early partial weightbearing and range of motion may be safe and not compromise the tendon transfer. In comparison, ISF was less robust. Although similar tendon displacement was observed, two early failures occurred during cyclical loading. Ultimate load to failure was inferior to the TT repair technique, indicating that more caution is required in the postoperative period if ISF is employed. As ISF in the foot gains popularity clinical research is required to determine outcomes compared with the traditional TT technique.
